# School-based HPV vaccination program implementation in municipalities of the São Paulo State, Brazil, from 2015 to 2018

**DOI:** 10.1590/0102-311XEN127423

**Published:** 2025-02-28

**Authors:** Roberta de Oliveira Piorelli, Helena Keiko Sato, Nubia Virgínia D’Ávilla Limeira de Araújo, Patrícia Emília Braga, Karina Takesaki Miyaji, Ana Marli Christovam Sartori

**Affiliations:** 1 Faculdade de Medicina, Universidade de São Paulo, São Paulo, Brasil.; 2 Divisão de Imunização, Secretaria Estadual de Saúde de São Paulo, São Paulo, Brasil.; 3 Instituto Butantan, São Paulo, Brasil.; 4 Centro de Referência para Imunobiológicos Especiais, São Paulo, Brasil.; 5 Hospital das Clínicas da Faculdade de Medicina, Universidade de São Paulo, São Paulo, Brasil.

**Keywords:** Human Papillomavirus Viruses, Human Papillomavirus Recombinant Vaccine Quadrivalent, Types 6, 11, 16, 18, Immunization Programs, Schools, Papillomavirus Humano, Vacina Quadrivalente Recombinante contra HPV tipos 6, 11, 16, 18, Programa de Imunização, Instituições Acadêmicas, Virus del Papiloma Humano, Vacuna Tetravalente Recombinante contra el Virus del Papiloma Humano Tipos 6, 11 , 16, 18, Programas de Inmunización, Instituciones Académicas

## Abstract

4vHPV vaccine was introduced into the Brazilian National Immunization Program in 2014, with vaccination at schools and healthcare facilities. Data on HPV vaccination program implementation in Brazil are scarce. This cross-sectional, exploratory study aimed to get a better understanding of the HPV vaccination actions and barriers to implement the school-based vaccination in municipalities of the São Paulo State, from 2015 to 2018, from the point of view of people who were responsible for conducting the actions. In November 2018, a questionnaire was sent to the state’s regional surveillance groups to be answered by people responsible for the vaccination actions in the municipalities. The questionnaire consisted of six open questions on HPV vaccination actions taken by the municipalities, from 2015 to 2018, including whether the school-based vaccination had been implemented or not, a program description, the barriers to implement it, how the program was evaluated, and the municipality plans regarding HPV vaccination program in the following years. 233 (36.1%) of the 645 municipalities answered the questionnaire. Most of them implemented both education or vaccination actions at schools. Reported barriers were health human resources shortage, education staff concerns on vaccination within schools, and students’ and parents’ unfamiliarity with HPV vaccination. Raising awareness on HPV infection and prevention among students and parents, ensuring appropriate human resources, and building a partnership between the health and education sectors are critical to have a successful school-based program.

## Abstract (pt)

A vacina contra o 4vHPV foi introduzida no Programa Nacional de Imunizações em 2014, com vacinação em escolas e unidades de saúde. Dados sobre a implementação do programa de vacinação contra o HPV nas escolas, no Brasil são escassos. Este estudo transversal e exploratório buscou compreender melhor as ações de vacinação contra o HPV e as barreiras para a implementação da vacinação escolar nos municípios do Estado de São Paulo, no período de 2015 a 2018, sob a ótica dos responsáveis pela realização das ações. Em novembro de 2018, foi enviado um questionário aos grupos regionais de vigilância estaduais para ser respondido pelos responsáveis pelas ações de vacinação nos municípios. O questionário continha seis questões abertas sobre as ações de vacinação contra o HPV realizadas pelos municípios no período de 2015 a 2018, incluindo se a vacinação escolar havia sido implementada ou não, uma descrição do programa, as barreiras para implementá-lo, como o programa foi avaliado e os planos do município para o programa de vacinação contra o HPV nos próximos anos. Dos 645 municípios, 233 (36,1%) responderam ao questionário. A maioria deles implementou ações de educação ou vacinação nas escolas. As barreiras relatadas foram escassez de recursos humanos em saúde, preocupações da equipe de educação com a vacinação nas escolas, e alunos e pais não familiarizados com a vacinação contra o HPV. Aumentar a conscientização sobre a doença e a prevenção do HPV entre alunos e pais, garantir recursos humanos adequados e construir uma parceria robusta entre os setores de saúde e educação são essenciais para um programa de vacinação escolar bem-sucedido.

## Abstract (es)

La vacuna contra el 4vVPH se incluyó en el Programa Nacional de Inmunización brasileño en 2014, con vacunación en escuelas y centros de salud. Los datos sobre la implementación del programa de vacunación contra el VPH en escuelas de Brasil son escasos. Este estudio transversal y exploratorio se propone comprender las acciones de vacunación contra el VPH y las barreras para la implementación de la vacunación en escuelas en los municipios del estado de São Paulo, en el período de 2015 a 2018, desde la perspectiva de los responsables de llevar a cabo las acciones. En noviembre de 2018, se envió un cuestionario a los grupos regionales de vigilancia de los estados para ser respondido por los responsables de las acciones de vacunación en los municipios. El cuestionario contenía seis preguntas abiertas sobre las acciones de vacunación contra el VPH llevadas a cabo por los municipios en el período de 2015 a 2018, relacionadas a si se había implementado o no la vacunación escolar, una descripción del programa, las barreras para implementarla, cómo se evaluó el programa y los planes del municipio para el programa de vacunación contra el VPH en los próximos años. De los 645 municipios, 233 (36,1%) respondieron al cuestionario. La mayoría de ellos implementaron acciones de educación o vacunación en las escuelas. Las barreras reportadas fueron la escasez de recursos humanos en salud, las preocupaciones del equipo educativo con la vacunación en las escuelas, y los estudiantes y padres que no están familiarizados con la vacunación contra el VPH. Una mayor concientización entre los estudiantes y sus padres sobre la enfermedad y la prevención del VPH, la garantía de recursos humanos adecuados y la creación de una sólida asociación entre los sectores de la salud y de la educación son fundamentales para que el programa de vacunación escolar sea exitoso.

## Introduction

The quadrivalent HPV vaccine (4vHPV) was introduced into the Brazilian National Immunization Program (PNI, acronym in Portuguese) in 2014 ^1^. At first, the program targeted girls aged 11 to 13 years and then was progressively expanded to a broader age range (9 to 14 years), a gender-free program (since 2017), and to also include immunocompromised young adults. Nowadays, 4vHPV vaccination is freely available for girls and boys aged nine to 14 years (in a single-dose regimen, since 2024 ^2^ and for women and men living with HIV/AIDS, solid organ and hematopoietic stem cell transplants, cancer, or using immunosuppressive therapy, aged 9 to 45 years old (in 3-dose regimen).

School-based vaccination strategy in both public and private schools during extramural actions was adopted, with flexibility to adapt to municipal reality. The vaccine was also made available in all public vaccination clinics in the country throughout the year. The first vaccination campaign at schools, in March 2014, was successful. The national coverage of the 4vHPV first dose (D1) was 94.4%, and 87% of Brazilian municipalities reached the recommended target coverage (80%), which was attributed to the school-based strategy ^3^ ^,^ ^4^. However, the national coverage of the second dose (D2) was just 40.8% and only 32% of municipalities reached the target coverage ^3^ ^,^ ^4^. In the following years, the strategy changed from school-based to vaccination predominantly in primary healthcare facilities and the country had trouble to achieve high 4vHPV vaccine coverage, particularly for D2, for boys, and in the North Region ^4^ ^,^ ^5^ ^,^ ^6^. Access barriers, fear of adverse events, failures in recording administered doses, and fake news have been blamed for the low vaccine coverage ^7^.

São Paulo State performed two school vaccination campaigns in the first year of the program (March and September) reaching D1 coverage > 100% ^3^. However, shortly after the second campaign has been started, in September 2014, a cluster of neurological events temporally associated to 4vHPV vaccination in a school in Bertioga, a city on the São Paulo State coast, was reported and was widely spread in traditional and social media ^3^. The young women fully recovered, and the cluster investigation concluded the events were likely immunization stress-related response (ISRR), but D2 coverage decreased to 44.6% in the state, despite vaccination at schools ^3^. Although the causes of this decrease in 4vHPV vaccine coverage are not completely clear, the widespread disclosure of neurological events in social media probably had a role. 

In Brazil, routine immunization is traditionally conducted almost exclusively at healthcare facilities with extramural actions, including schools, conducted during vaccination campaigns. The vaccination strategy responsibility rests on municipalities and data on the school-based HPV vaccination program implementation are scarce ^8^. Considering this first Brazilian experience with school-based routine vaccination and the HPV vaccine specificities, it is important to have a better knowledge on the implementation. This study aimed to describe the HPV vaccination actions, strategies, barriers, and facilitators to implement the school-based vaccination program and proposed alternatives in São Paulo municipalities from 2015 to 2018.

## Methods

This was an observational, transversal, and descriptive study based on a questionnaire produced by the Immunization Division of Epidemiological Surveillance Center of Disease Control Coordination Office of the São Paulo State Health Department (DI/CVE/CCD/SES-SP, acronym in Portuguese), in November 2018. The questionnaire was sent to the state Regional Surveillance Groups (GVEs, acronym in Portuguese), requesting them to send it to all municipalities in their region, to be answered by the people responsible for the municipality vaccination actions and to return the answers to DI/CVE/CCD/SES-SP. The purpose was to conduct a Rapid Assessment, i.e., an exploratory study to get a preliminary understanding of HPV vaccination strategies adopted in the state, their scope, barriers, and facilitators to implement the school-based vaccination program, from the point of view of those who performed the actions.

São Paulo State, in the Southeast of Brazil, has 46 million inhabitants, distributed in 645 municipalities and 28 GVEs.

The questionnaire consisted of six open questions on HPV vaccination actions taken by the municipalities, from 2015 to 2018, including whether the school-based vaccination had been implemented, a description of the actions taken, the scope, the barriers to implement it, how the actions had been evaluated, and the municipality plans for the HPV vaccination program in the following years. 

The answers were sent to DI/CVE/CCD/SES-SP in November-December 2018, in quite heterogeneous format. Some municipalities sent individual answers, whereas some GVEs assembled their municipalities answers to forward to DI/CVE/CCD/SES-SP, and in this case, it was not always possible to identify which municipalities implemented an action, or even which ones answered the questionnaire. Whenever possible to distinguish individual municipality answers among the GVEs’ assembled answers, these were distinguished and analyzed individually. The answers were analyzed and assembled into groups, which were created after reading all the answers.

The actions implemented in schools were classified as “education” and “vaccination”. Any action aimed at providing guidance on HPV infection and vaccination, alerting on vaccination campaigns and places where 4vHPV vaccine would be available and answering students and parents’ questions were classified as Education actions. Actions targeting the vaccination itself were classified as Vaccination actions, independently of whether it occurred in schools or in healthcare facilities, if it involved schools in some way. 

The reported barriers were classified as difficulties related to school, health, parents and students, and other factors. School-related barriers were defined as those related to school employees, such as the teachers, support staff, and managers at the local level, such as coordinators and principals, or at higher level, such as government bodies and departments, regarding their support, understanding, and acceptance of actions. In addition, issues related to the school’s physical space and environment were also classified in this group. Health-related barriers were those related to healthcare workers at all levels, physical space, and available resources, such as human resources, vaccine doses and supply, and transport of professionals and materials. Parents and students-related barriers referred mostly to their lack of interest or even resistance to HPV vaccination or education actions proposed at schools. Barriers related to other factors include any other reported difficulties that could not be assembled in the previous ones, such as news published by the media. The municipalities' positive experiences with school-base HPV vaccination program were also reported.

HPV vaccination plans for the following years were separated into actions related to education or vaccination, according to considerations used to classify the conducted actions described above. 

Whenever possible, the results obtained from assembling the answers were shown with absolute and relative frequencies, separated into municipalities whose answers could be individualized and those whose answers were assembled by the respective GVEs. The difficulties reported by municipalities with individualized answers were described separately for municipalities that conducted any action at schools and those that did not. 

This study was approved by the Ethics Committee of Clinics Hospital of Faculty of Medicine of the University of São Paulo, (HC-FMUSP CAPPesq, CAAE 17905219.0.0000.0065) on August 22nd, 2019. Anonymous data were used.

## Results

The DI/CVE/CCD/SES-SP received individual answers from 167 municipalities and answers assembled by eight GVEs that correspond to 282 municipalities. The analysis of the GVEs assembled answers suggested probably 66 of the 282 municipalities answered the questionnaire and 40 did not answer. It was not clear if the other 176 municipalities in those GVEs answered or not. So, 233 (36.1%) of the 645 São Paulo’s municipalities provided an answer, either individually (167) or assembled by the GVEs (66), and 236 (36.6%) did not answer. 

Among the 233 municipalities that answered the questionnaire, 200 (85.8%) reported having implemented some HPV vaccination action at schools (education or vaccination), from 2015 to 2018, and 32 (13.7%) had not implemented any action at schools (one municipality did not answer). Among those 144 municipalities that sent individual answer and had implemented any action, 65 (45.1%) had implemented both HPV education and vaccination at schools, whereas 64 (44.4%) had implemented only vaccination and 13 (9.1%) only education (two municipalities did not answer). Among the municipalities that detailed their actions scope, more than half reported covering municipal, state, and private schools.

Box 1 presents the education and vaccination actions most frequently reported by the municipalities. 

**Box 1:** HPV education and vaccination actions implemented at schools by municipalities of the São Paulo State, Brazil, from 2015 to 2018.

TYPE OF ACTION	DESCRIPTION
Education	Awareness letter on HPV infection and vaccine sent by schools to parents or guardians Lectures on HPV at schools for parents and guardians Classes and other activities (chat groups, theatres) to raise awareness on HPV among students Availability of educational/informative materials Training on HPV infection and vaccination for teachers and principals Sensitization of professionals of the primary healthcare network Disclosure of HPV infection and vaccination in the media and social networks
Vaccination	School-based vaccination Active searching of unvaccinated students referring them to be vaccinated at the health services Checking vaccination status Preparing and sending vaccination refusal or consent forms to be signed by parents/guardians (in case of vaccination at school) Extending the opening hours of healthcare facilities during the vaccination campaign School requirements to the health services to check the students’ vaccination status and issue a certificate of updated vaccination Requirement of a certificate of updated vaccination provided by the health services on students’ enrolment at schools

Several municipalities reported joint actions of the health and education professionals targeting both parents and students. Some established a partnership between the municipal health and education departments to conduct health education for teachers and principals or included HPV in the science classes, with support material prepared by the Municipal Health Department. In other municipalities, health professionals participated in parent-teacher conferences at schools. Some teachers were trained to be a link between students and health professionals/services. Taking advantage of activities routinely conducted at schools, when most parents and students were present, made education actions easier.

Training teachers and community health agents as multipliers and spreading the importance of HPV vaccination in the media and social networks were also strategies used by some municipalities. A municipality made a partnership with a teenager youtuber to reach the target population for vaccination. 

Education staff support to school-based HPV vaccination facilitated to conduct the program. Conducting HPV vaccination campaigns at schools few days after the education activities improved students’ adherence to vaccination. Allowing the parents/guardians to be present at schools on the vaccination day or vaccinating the students at school entrance or exit times facilitated the action. Some municipalities suspended classes on the vaccination day. Others took advantage of preexisting events with the presence of parents/guardians at school to conduct the actions, which facilitated talking to and raising awareness among both parents/guardians and students. Some schools offered a snack after vaccination. The Family Health Strategy (FHS) teams’ involvement was a facilitator.

Some municipalities reported conducting additional actions simultaneously to HPV vaccination campaigns at schools, such as extending the opening hours of healthcare facilities for vaccination to cover students who missed school on the vaccination day, conducting house-to-house vaccination in urban and rural areas, or delivering flyers at the absent students’ homes, with a duplicate signed by parents/guardians (although this last action was considered not successful in increasing students adherence to vaccination). A municipality kept an emergency service available for possible adverse events supposedly attributable to vaccination or immunization (ESAVI). 

Several municipalities reported a consent form was sent to parents or guardians to allow their children to receive the HPV vaccine at school, whereas in others, a refusal form was sent to be signed only by parents or guardians who did not authorize their children to be vaccinated.

The students’ vaccination status evaluation by the health professionals involved in school-based vaccination actions, conducted in several municipalities, contributed to planning the campaign, and storing the necessary numbers of vaccine doses.

Alternative actions were implemented by some municipalities that did not conduct school-based vaccination. Most frequently, referring students by schools to be vaccinated at healthcare facilities. Requirement of a certificate of updated vaccination, issued by the health services, on school enrollment was adopted by several municipalities. 

Some municipalities observed actions at schools led to an increase in the demand for HPV vaccine and for updating other vaccines at healthcare facilities.

### Barriers faced by municipalities to conduct school-based HPV vaccination

Most municipalities reported barriers to school-based vaccination. Both municipalities that conducted school-based HPV vaccination and those who did not implement it reported similar difficulties. Box 2 presents the most frequently reported barriers.

**Box 2:** Most frequent barriers to school-based HPV vaccination faced by municipalities of the São Paulo State, Brazil, from 2015 to 2018.

TYPE OF BARRIERS	DESCRIPTION
Related to schools and the Education Department	Education professionals’ concerns on school-based vaccination Lack of partnership between the Education and Health sectors Education staff unfamiliarity on HPV infection and vaccine Some school staff beliefs leading to resistance to HPV vaccine Education professionals’ concerns of vaccine-associated adverse events occurrence within school Education professionals’ fear of school routine disruption Lack of permission for school-based vaccination from the Education Department Inadequacy of schools’ physical structure for vaccination
Related to health services and the Health Department	Shortage of human resources for vaccination at schools Lack of transport for the health teams and supplies Insufficient vaccine doses Competing public health priorities, mainly simultaneous vaccination campaigns in response to outbreaks
Related to parents/guardians and students	Students’ fear of vaccination Parents/guardians and students concerns on vaccine-associated adverse events Students’ absenteeism Return of signed consent form by parents/guardians Vaccination refusal by parents/guardians Unavailability of vaccination cards Parents/guardians’ fear that HPV vaccine would encourage their children to start their sexual life earlier Unacceptance of school-based vaccination Parents/guardians and students lack of knowledge on HPV infection and vaccine and its importance Parents/guardians and students lack of interest on HPV education and vaccination actions
Other	Disclosure of negative content and misinformation on HPV vaccine by the media and social networks Disclosure of adverse events supposedly attributable to HPV immunization by the media and social networks Controversies surrounding a vaccine associated to a sexually transmitted infection

School-related barriers frequently reported by the people who were responsible for vaccination in the municipalities were the lack of partnerships and joint actions between Health and Education staff, the lack of involvement of the Education Department in diseases prevention projects, the lack of interest of education professionals and difficulties in raising awareness among teachers and principals that did not take HPV vaccination as a priority. School professionals’ resistance to conduct any health procedures at school or to ask students to bring their vaccination cards were reported. Some schools’ staff did not want to get involved and just delegated the approach to the topic and related activities to the health professionals. 

Some education staff believed school-based vaccination is not appropriate due to possible occurrence of adverse reactions and changes in school routine. Principals and teachers were afraid that the school-based vaccination would bring disorders, disrupting the school routine and the classes progress. Some principals and teachers argued that “school is not a place to feel pain”. 

The inadequacy of the school’s physical structure for the vaccination procedures and lack of structure to deal with immunization stress-related response were pointed out by municipalities.

Some municipalities reported greater resistance from the private schools; some of them did not accept HPV vaccination, such as a religious school.

Logistics difficulties were reported, such as: adjusting the hours of the health teams to the school hours, sending a vaccination communication or the consent/refusal term to parents/guardians, delay of the schools to deliver a list of the target students to vaccination, or the lack of disclosure of the school year calendar for properly actions planning. Some school units were not properly organized, jeopardizing the actions.

Scarcity of human resources was the most frequent health-related barrier, reported by several municipalities. Healthcare workers scarcity, which was a limiting factor even before school-based vaccination implementation, in addition to the high demand of the school-based program seemed to be the greatest health-related barrier. Some municipalities mentioned having trouble organizing teams to conduct the vaccination at several schools, several times a week. Involving the FHS teams helped, but this program does not always cover the entire municipality, making actions in some places difficult. Competing public health priorities, such as the yellow fever outbreak response actions (2016 to 2018), were also reported to hamper school-based vaccination.

Including boys in HPV vaccination program three years after the girls was another source of difficulties. Since schools usually separate students by age, schools or classes whose girls had already been vaccinated needed to be revisited to vaccinate the boys, increasing the demand for health workers. Students’ absenteeism on the vaccination days was another reason for having to return several times to schools. 

At least two cities mentioned difficulties in acquiring enough HPV vaccine doses to reach the entire target population. Other had troubles in registering the vaccination conducted at schools, resulting in unreliable data.

Students’ fear of needles, both parents and students concerns on vaccine-associated adverse events, parents refusal, not returning the consent form, parents/guardians and students lack of interest in education activities on the topic, considered a taboo, lack of knowledge on HPV infection and vaccine, and parents lack of commitment on their children immunization were also reported to hamper actions at schools.

Convincing some parents/guardians, mainly of girls, that HPV vaccination aims to prevent HPV-related cancers and not to encourage the adolescent’s early sexual debut was a commonly reported difficulty. Some parents felt their children were too young to be aware of HPV infection and its prevention. Lack of concern about HPV infection was also noted. Some parents/guardians did not accept the school-based HPV vaccination, and some of them put pressure on the school principals to prevent health professionals from entering the school. 

A municipality reported that “vaccination at schools was tumultuous and many teenagers made jokes on their colleagues”. Another reported some nine-year-old children had hypotension and syncope after vaccination.

Municipalities that referred the students to receive HPV vaccine at the health facilities also reported adolescents’ low adherence to this strategy and access issues faced mainly by full-time students. 

Negative news on HPV vaccine disclosed by the media were cited as harmful to the vaccination actions in some municipalities. Spreading news on events supposedly attributable to vaccination caused stress and reduced students’ adherence to the second dose vaccination. Religious issues also hampered HPV vaccination actions.

Despite the reported difficulties, most of the municipalities that implemented school-based HPV vaccination reported the program had positive results. Most had plans to implement HPV education or vaccination actions at schools in the following years. Most municipalities that had conducted school-based vaccination in the studied period intended to keep it or to expand its coverage. Some municipalities that did not implement school-based vaccination were planning to do so in the following years, whereas others were planning to conduct HPV vaccination at the healthcare facilities, with active search of unvaccinated children/adolescents and requirement of an updated vaccination certificate issued by the health service on school enrollment. 

## Discussion

This study allowed to quickly develop a preliminary understanding of the status of school-based HPV vaccination in São Paulo municipalities, from the point of view of the managers of vaccination actions in these municipalities. 

School-based or mixed (both at schools and at health facilities) strategies usually reach higher vaccination coverage among adolescents than health facility-based vaccination ^5^ ^,^ ^9^ ^,^ ^10^. School-based vaccination is adopted by some of the most successful HPV vaccination program worldwide, such as Australia ^11^ and United Kingdom ^9^. In England, all vaccines recommended to adolescents (HPV, meningococcal ACWY and tetanus, diphtheria and polio [dT/IPV]) are delivered via school-based program ^9^. Implementing school-based HPV vaccination may be an opportunity to build or strengthen school and adolescent health ^12^. Besides adopting school-based vaccination program, Australia, Mexico, and Peru, which successfully achieved high HPV vaccine coverage, used social marketing techniques in their vaccination campaigns to better engage the target population ^13^.

Before 4vHPV introduction into the Brazilian PNI, a demonstrative project of school-based HPV vaccination targeting girls aged 10-16 years at both public and private schools in Barretos (São Paulo State), in 2010-2011, reported favorable results. High vaccine coverage (> 85%) was achieved among the target population with low dropout rate (97.2% of those who started vaccination completed the 3-dose regimen) ^8^. 

In Indaiatuba (São Paulo State), school-based HPV vaccination was implemented in 2018. In the first year, it targeted 5,600 girls and boys aged 9-10 years, whereas vaccination of adolescents aged 11-14 years was conducted at health services. The number of 4vHPV first dose administered to all ages increased 78% as compared to the previous year. Among children aged 9-10 years, 4vHPV coverage increased from 16% (2017) to 50.5% (2018). However, in the second year of the program, the first dose coverage among the target children dropped to 26.9% ^6^. Lack of interest of the involved staff and other public health priorities (yellow fever outbreak and measles reemergence in São Paulo, in 2018 and 2019, respectively, both triggering large vaccination campaigns), which competed for human resources, jeopardized the school-based vaccination program ^6^. Shortage of human resources, a preexisting issue in the public health system, was the most common health-related barrier identified in our study. Ensuring appropriate human resources is critical to having a robust school-based program.

Education professionals’ concerns or lack of commitment on school-based vaccination were barriers commonly reported by the people who were responsible for the vaccination actions in São Paulo municipalities. School-based vaccination requires a great involvement of both health and education professionals at all levels. Teachers are a key component of the communication strategy ^10^. Building the health and education sectors partnership is critical.

Lack of knowledge of parents and students on HPV infection and the vaccine, and concerns on vaccine safety were identified in our study as main barriers to the program. A survey, in São Paulo city, in 2015-2016, found adolescents had low knowledge on HPV and HPV vaccine. Girls were better informed than boys and the main source of information for adolescents was school (for 39% of them), explained by the fact that the study was conducted a year after the first HPV vaccination at schools, when HPV education actions were conducted in many schools ^14^. Other survey in Ouro Preto (Minas Gerais State) showed both parents and students had low knowledge on HPV, cervical cancer, and HPV vaccine ^15^. A study that analyzed HPV vaccination in Latin American countries also found limited knowledge on HPV and its consequences and misguided safety concerns, among others, as main barriers to HPV vaccination in the region ^16^. In our study, propagation of misinformation and negative news on HPV on the media and social networks was also reported to impact students’ adherence to vaccination. Spreading correct information and raising awareness are important for an HPV vaccination program ^10^. A study in Texas (United States), showed educating parents/guardians, school staff, and health professionals increased HPV vaccine uptake ^12^. 

School absenteeism and not returning the consent form, frequently reported in our study, were also identified by Australian stakeholders as major barriers to their school-based vaccination program ^11^. Some strategies were suggested to deal with this issue, such as: using electronic consent forms, to remove reliance on students taking home and returning paper forms, resulting in unreturned forms by phone call, email, letter, or SMS to parents; verbal parental consent obtained on vaccination day; and allowing mature students to consent for themselves with endorsement of the principal ^11^. The Brazilian PNI/Ministry of Health recommended using a refusal term, distributed by schools before HPV vaccination to be signed only by those parents/guardians who did not authorize their children vaccination ^17^. The refusal term simplifies the process and was adopted by some São Paulo municipalities, such as Indaiatuba, where 7.5% of children who were target for vaccination at schools showed a refusal term signed by parents, in 2018 ^6^. However, some school professionals’ concerns on not having an authorization signed by parents was an issue.

This study has several limitations. First, the questionnaire had open questions and some of them were not well written and had different interpretation by respondents. Second, in the answers assembled by some GVEs, we were not always able to identify how many or which municipalities answered the questionnaire. Third, Brazil is a huge country with great regional differences both socioeconomic and in the health and education services structure, so our findings may not apply to other regions/states. Furthermore, the COVID-19 pandemic disrupted the routine health services and led to schools shutting down and the interruption of the schools’ immunization programs. The current scenario may be quite different from the pre-pandemic.

A sharp reduction in the number of 4vHPV administered doses has been reported during the pandemic in Brazil ^18^. Since 2022, several municipalities/states are planning to resume the school-based HPV vaccination, considered by managers as strategic to reach the target population and increase HPV vaccine coverage. In 2023, PNI/Ministry of Health adopted Microplanning for High-Quality Vaccination Activities, at municipalities level, as a methodology to assist in organizing vaccination actions and recovering vaccination coverage ^19^. Reporting the actions, barriers, and facilitators as described by the health professionals who were responsible by the actions in São Paulo state municipalities that implemented the program may help managers who are planning to do so or to improve the existing programs.
